# Academy of Stomatology

**Published:** 1905-05

**Authors:** 

**Affiliations:** Academy of Stomatology


### ACADEMY OF STOMATOLOGY.

A REGULAR meeting of the Academy of Stomatology of Philadelphia was held at its rooms, 1731 Chestnut Street, on the evening of Tuesday, November 22, 1904, the President, Dr. I. N. Broomell, in the chair.

A paper was read by Dr. M. I. Schamberg, of Philadelphia, entitled "The Etiology, Pathology, and Treatment of Troublesome Tooth Sockets."

(For Dr. Schamberg's paper, see page 217.)

### DISCUSSION.

*Dr. H. B. Hickman.*—I would like to recall a case which I reported here some time ago, and which has relation to this paper. The case was one in which it was necessary to remove the lower right second bicuspid to relieve pain, even after the tooth had been opened through the apical foramen and left open. I found the root-canal had been filled for ten years. The tooth was extracted by a specialist who was very careful, both before and after the operation, in the use of antiseptics. On the day following the extraction the pain was very severe, and worse when the patient was lying down. Cocaine, morphia, and sulphate of zinc were tried, but carbolic acid alone gave relief. Osteomyelitis developed, and the patient died as the result of this troublesome tooth socket.

*Dr. James Truman.*—I have not heard any paper in recent years that has impressed me more than this paper of Dr. Schamberg; not that we have in it anything especially new, and I do not presume that he gives it with that idea, but it is the first time I have ever heard a paper that really took the whole matter in hand and gave us an intelligent conception of the duty of the dentist.

I do not know that I have any criticism to make, except perhaps with reference to his statement of the necessity to remove a tooth. If we are to remove all teeth that are in a diseased condition, I am fearful that we will not keep any when an alveolar abscess, so-called, has developed, for not one of those teeth could be considered healthy. All have a large amount of necrotic tissue at the apex or apices of roots and are liable to produce the so-called gum-boil at any time. I would not want to extract all those teeth.

Upon the whole, I do not quite agree with his view that it is best in the first instance to use hydrogen dioxide. Hydrogen dioxide is, in my opinion, not the harmless agent it has been supposed to be, and by injecting it into tooth sockets there is a possibility of producing trouble. I have seen cases in the clinics in which serious conditions have resulted.

I would like to ask Dr. Schamberg what he would do in a case of abscess when he allows the coagulum to fill up and the patient thereafter suffers intensely from pain of extraction. I do not think it is a good thing to have that coagulum remain without treatment. It seems to me that the socket should be curetted out and antisepticized. I think that spraying with hydronaphthol is more irritating than with phenol sodique. Why the latter should be objectionable in a tooth socket when it is so advantageous in other directions I do not understand. In a general way I think I am in harmony with the treatment given to tooth sockets.

I agree thoroughly with his concluding sentence,—that a man who undertakes to extract teeth and does not make his hands and instruments thoroughly sterile, and does not make the mouth sterile subsequently, does not understand his professional duty to his patient.

*Dr. M. H. Cryer.*—I am much pleased, and came in from the country purposely to hear Dr. Schamberg's paper.

I would not care to recommend the use of hydrogen dioxide in tooth sockets after extraction, especially in the lower jaw, as the cancellated tissue is so open that when the medicament comes in contact with either pus or blood, oxygen is freely liberated with almost an explosive effect, so that it may force septic matter, if it be present, into the cancellated tissue. Dr. Woodward has just reminded me of a patient that was referred to me for treatment. A diseased lower second bicuspid having an abscess around the root had been extracted; the necrotic socket was treated with hydrogen dioxide for some time, and when I made an examination I could pass an ordinary silver probe from the enlarged tooth socket to the angle of the jaw, and forward to near the symphysis. My opinion is that the internal portion of the bone had been destroyed by the hydrogen dioxide in combination with pyogenic organisms. As soon as this medicament was discontinued and other treatment instituted, the patient had relief, and finally the space was filled with new tissue through granulation.

Hydrogen dioxide is not used by the surgeon as in former years, especially in deep-seated wounds. It is useful in surface wounds, however, when carefully applied, and I have no doubt that, with the care and judgment Dr. Schamberg would give, a tooth socket could be wiped out with hydrogen dioxide upon a piece of cotton.

*Dr. William H. Trueman.*—There is food for thought in the paper read. There can be no question but that some cases of alveolar abscess are to some patients a serious menace, because cases are on record where they have been followed by serious consequences. So it is with various other diseases usually considered trivial, especially if they are not properly treated. There is no doubt but that in some cases the teeth had better be extracted. We should not, however, consider alone the *possibility* of serious injury, but the *probability.* The danger from alveolar abscess is, under ordinary circumstances, exceedingly slight. I have had six teeth in my own mouth which have abscessed now and again; first one and then another causes me considerable pain and inconvenience, notwithstanding, I have no fear of serious consequences, and would suffer a great deal rather than have them extracted. One case in my experience made a strong impression upon my mind: A gentleman came to me early in my practice with a central incisor badly abscessed. It had been treated unsuccessfully by several dentists. There was a copious discharge of foul-smelling pus from a fistula far back of the roof of the mouth, near the termination of the hard palate, and another through the gum over the apex of the root, and it seemed to be a hopeless case. It was a front tooth, and, as I did not care to spoil his good looks, I attempted treatment. This was successful in so far that in a short time the discharge became very little, and instead of pus was simply a serum. This has continued for about thirty years. Although the fistula on the roof of the mouth seems to have closed, he can now, by making pressure upon the roof of his mouth with his tongue, cause a little serum to ooze out through the fistula on the gum over the root. The gentleman may have taken a very serious risk in retaining that tooth, but the risk has not materialized. Deciding these questions calls for great care and judgment. It is a very serious matter to deprive a patient of a tooth that may be useful, especially one that must be replaced by a substitute.

Another case I recall is of a gentleman who had a badly diseased bicuspid. It had caused an abscess, and around the fistula the tissues had enlarged until they formed a tumor the size of a large marble. He told me it had been in much the same condition several years. I told him it should be removed promptly, but he treated the advice lightly, and said he would have it done at some convenient time. I wrote him a long letter that evening, drawing a very vivid picture of the possibilities if it were left in. I heard nothing from him for about a year, when I again examined his mouth. The tooth was still there, its surroundings quite normal, and there was nothing to show that it had been abscessed, and he assured me it had had no treatment whatever. Had he followed my advice he would have lost a useful tooth. We have to remember the peculiarities of individuals. Some people are susceptible to certain poisons; others are not. We meet with a very few cases, comparatively, that seem to point the moral now and again brought to our notice. These possibilities demand serious consideration; at the same time we must bear in mind not the possibility only, but also the probability.

*Dr. Louis Jack.*—I have very little to say beyond what has already been said by Dr. Schamberg. I have found from a large experience with hydronaphthol, 1 in 300 parts, that it is entirely non-irritating.

With reference to abscess of the lower third molar, there is danger that the pus will not find its way through the external side, and is inclined to pass inward on account of the less resistance in this direction; therefore such cases require the closest attention. I remember at one time being called upon by a prominent clergyman for an inflamed third molar, which I ordered out. He replied that he would not have it removed. Subsequently I was called to see the patient, who was dangerously ill on account of the condition of the throat. I stated that the tooth should be immediately removed, as I was afraid pus had already begun to burrow into the hyoid tissues. The physician asked me to delay twenty-four hours, but I felt that by so doing the patient's life would be in danger, and my counsel was carried out. The patient was in bed for several weeks in consequence of the pyæmic condition already existing.

I remember a similar case of a patient in the upper part of the city. The patient died. The physician's diagnosis had been malignant quinsy. The post-mortem revealed excessive infiltration at the base of the tongue; even the hyoid bone was infiltrated with pus.

For the relief of pain in a dry socket following extraction I have found most satisfactory a suppository composed of orthoform, vaseline, and oxide of zinc. The pain of a dry socket I consider more severe than almost any other form of toothache, and it is continuous.

When a patient is sent to me immediately after extraction of a tooth, my usual practice is to spray the parts either with acetanilide or with the normal aqueous solution of hydronaphthol.

*Dr. P. B. McCullough.*—It does not always happen that post-operative treatment, other than that which can be done at the doctor's office, can be carried out. This is true in the majority of cases. It is useless, therefore, to recommend a thing, however valuable, if it cannot be practised. To that end, I have for a number of years prescribed salt, advising the patient to rinse the mouth frequently with a strong salt solution. Salt does not seem to be regarded as possessing very much antiseptic property, and I have not seen any reference to it, except in a newspaper article stating that sugar and salt were mild antiseptics.

With reference to the use of hydrogen dioxide, I have observed the use of this followed by washing with a dilute solution of carbolic acid. To my inquiry why one antiseptic should be followed by another, it was stated that the moment hydrogen dioxide decomposed it ceased to have any beneficial effect, and that, in order to remove what remained of its decomposition, it was to be followed by the use of an unchangeable antiseptic.

*Dr. Schamberg* (closing).—It is not unusual to read in the dental journals from month to month accounts of fatal cases following tooth-extraction. Some of these are directly traceable to negligence, others are undoubtedly due to predisposing conditions of the patient.

Regarding the case referred to by Dr. Hickman, there is some question in my mind whether death was due to osteomyelitis, or whether the affection of the tooth socket was a result of the lowered state of the patient's vitality. The eruption upon the man's body may have been of septic origin, but in the absence of an autopsy it cannot be positively stated that death resulted from the jaw lesion. It is unusual for a tooth socket to give such serious trouble when the tooth is removed without difficulty and with aseptic precautions, there being no pronounced local disturbance and the patient being otherwise in good health.

Believe me, I do not encourage the removal of troublesome teeth because I make extraction a specialty. The point I wish to bring out is that there is an ultra conservatism at the present time which says, "Keep a tooth as long as you can." Teeth so retained are often a menace to health and barriers to proper mastication. I therefore believe that such teeth, unless promptly cured, should be removed. I also believe that a properly adjusted artificial tooth will render better service than an irritable tooth which interferes with mastication.

I agree with Dr. William Trueman that many people keep uncured abscessed teeth in their mouths for many years. I believe that such people make a great mistake. Dr. Trueman has said that we should consider the *probability* of trouble rather than its *possibility*. If that were the case, there would be some question as to whether it were wise for us to drink unboiled Schuylkill water, for many people who drink Schuylkill water as it comes from the spiggot never get typhoid fever. A proportion, however, who indulge in this indiscretion do acquire that disease, and the practice is, therefore, considered a hazardous one. By similar reasoning it becomes hazardous to retain uncured teeth the pus of which is likely to be absorbed in the mouth or gastro-intestinal tract.

I might mention, with the permission of one of our fellow-members present, the influence of an alveolar abscess upon his wife's general health. The abscess was of fifteen years' duration upon an upper lateral incisor tooth. It had persisted during that long period in spite of treatment directed through the canal of the tooth and conducted by several of our prominent dental practitioners. The doctor brought his wife to me for root amputation because he felt that the abscess had something to do with his wife's impaired health. The operation resulted not alone in a cure of the abscess, but in a marked improvement in the patient's health. This was evidently due to the complete elimination of the septic area surrounding the necrotic end of the root. I believe there are many patients who consult their physician for the treatment of systemic disorders which could more readily be eradicated by the dentist. Some patients suffer from gastro-intestinal disturbances and other constitutional ailments due to the absorption of pus generated in the mouth.

Dr. Jack spoke of the removal of third molars. These teeth are, as a rule, troublesome before, during, and after extraction. I had a patient sent to me recently with Ludwig's angina, there being so much swelling of the throat that the patient almost suffocated. The removal of a crowded wisdom-tooth promptly relieved the patient. This is but one of many conditions which may follow the prolonged retention of bothersome wisdom-teeth. Suppuration around these teeth is not so often due to putrescent pulps as to pus-pockets formed by overlapping gum tissue.

The suppository recommended by Dr. Jack is a very good one. My method of relieving painful sockets is to dip tampons of cotton into campho phenique, then into powdered orthoform, and thence into the socket. These are to remain for twenty-four hours and then renewed.

Dr. McCullough referred to the impracticability of poor people following the post-operative treatment outlined by me. I supply most of my patients with small bottles of mouth-washes to be used after extraction. The manufacturers of these preparations are glad to supply the profession with samples for distribution among their patients. When I learn that a patient has no mouth-wash at home, I supply him with one which is to be renewed at the drug store when exhausted.

The suggestion of Dr. McCullough, to follow the use of hydrogen dioxide by some other antiseptic, is a good one. This is my invariable practice.

Dr. Cryer's objection to peroxide of hydrogen I can only too readily agree with. I realize that a great deal of harm can be done by the injudicious use of this agent.

In speaking to Dr. Daland of the patient who suffered from boils as a result of an abscessed tooth, he expressed himself as being confident of the fact that pus lurking in the oral cavity and gaining access to the system, either by ingestion or absorption, is a factor to be taken into consideration when studying the etiology of furunculosis.

Otto E. Inglis,
*Editor Academy of Stomatology.*

